# Supplementary material for: Urinary Dialkylphosphate Metabolite Levels in US Adults—National Health and Nutrition Examination Survey 1999–2008
Source: Int J Environ Res Public Health. 2019 Nov 20;16(23):4605. doi: 10.3390/ijerph16234605 (PMC6926828; doi:10.3390/ijerph16234605)
Supplement: Supplementary file 1 [file ijerph-16-04605-s001.pdf]

Supplementary

Figure S1. Time trends in urinary DMP metabolites in adults from 1999 to 2008.

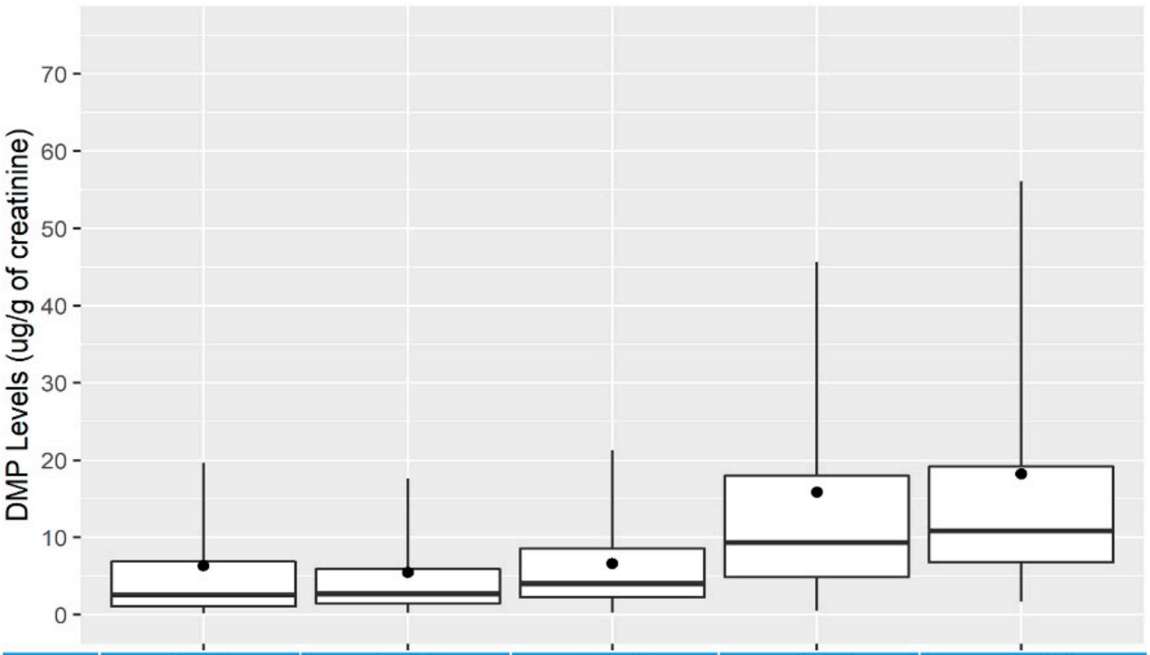

	1999-2000	2001-2002	2003-2004	2005-2006	2007-2008
Percent Above DL	51.14%	47.99%	50.99%	35.46%	38.11%
Maximum	203.39	85.71	57.10	265.81	327.15
95th Percentile	19.69	17.67	21.28	45.61	56.21
Mean	6.35	5.49	6.63	15.88	18.23
75th Percentile	6.87	5.92	8.55	18.02	19.18
Median	2.58	2.72	4.03	9.37	10.87
25th Percentile	1.05	1.46	2.25	4.90	6.79
Minimum	0.21	0.23	0.32	0.47	1.69

Boxplots represent the minimum, first quartile, median, third quartile, and 95th percentile values. Mean values are represented by the black circle.

Figure S2. Time trends in urinary DEP metabolites in adults from 1999 to 2008.

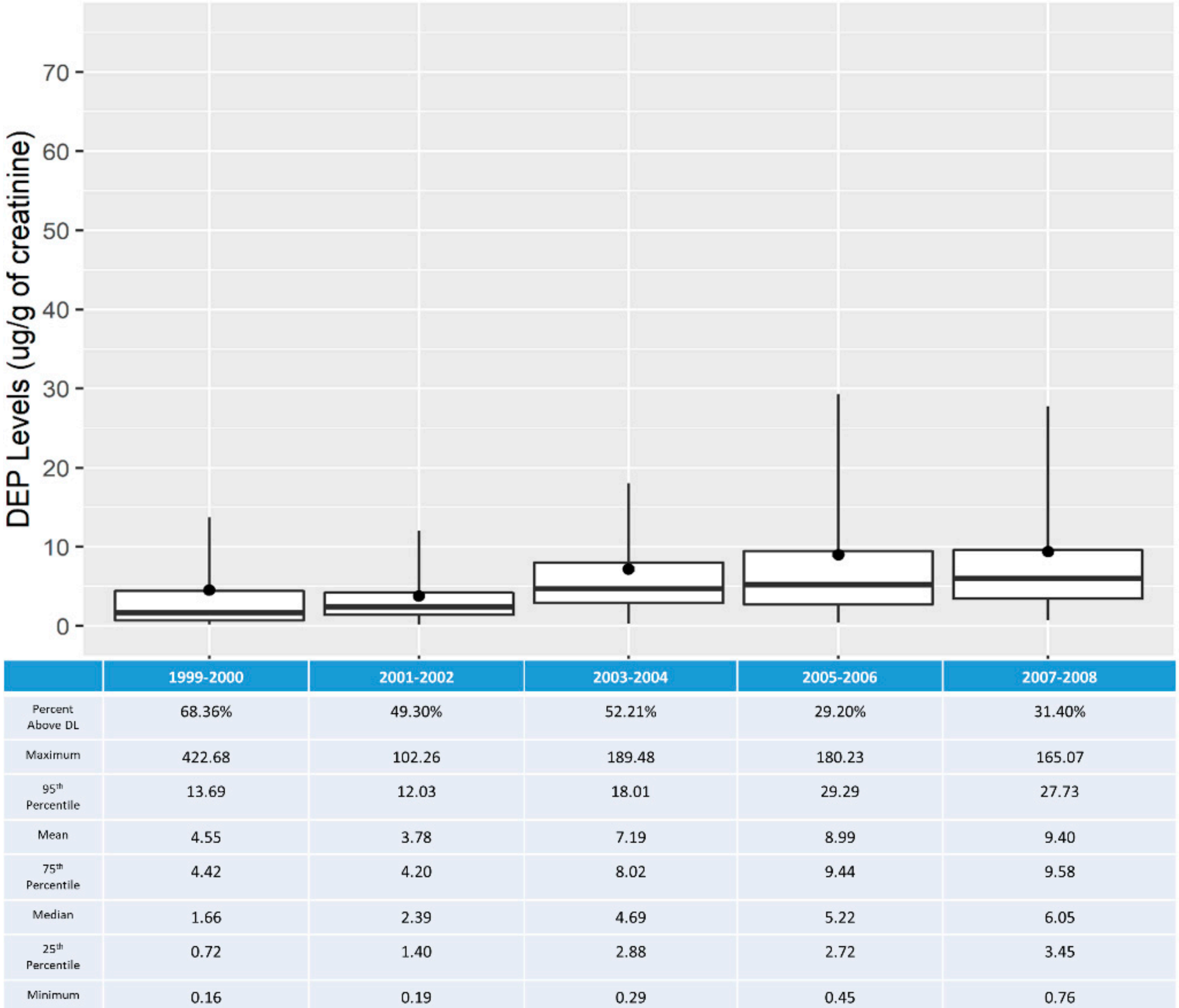

	1999-2000	2001-2002	2003-2004	2005-2006	2007-2008
Percent Above DL	68.36%	49.30%	52.21%	29.20%	31.40%
Maximum	422.68	102.26	189.48	180.23	165.07
95th Percentile	13.69	12.03	18.01	29.29	27.73
Mean	4.55	3.78	7.19	8.99	9.40
75th Percentile	4.42	4.20	8.02	9.44	9.58
Median	1.66	2.39	4.69	5.22	6.05
25th Percentile	0.72	1.40	2.88	2.72	3.45
Minimum	0.16	0.19	0.29	0.45	0.76

Boxplots represent the minimum, first quartile, median, third quartile, and 95th percentile values. Mean values are represented by the black circle.

Figure S3. Time trends in urinary DMTP metabolites in adults from 1999 to 2008.

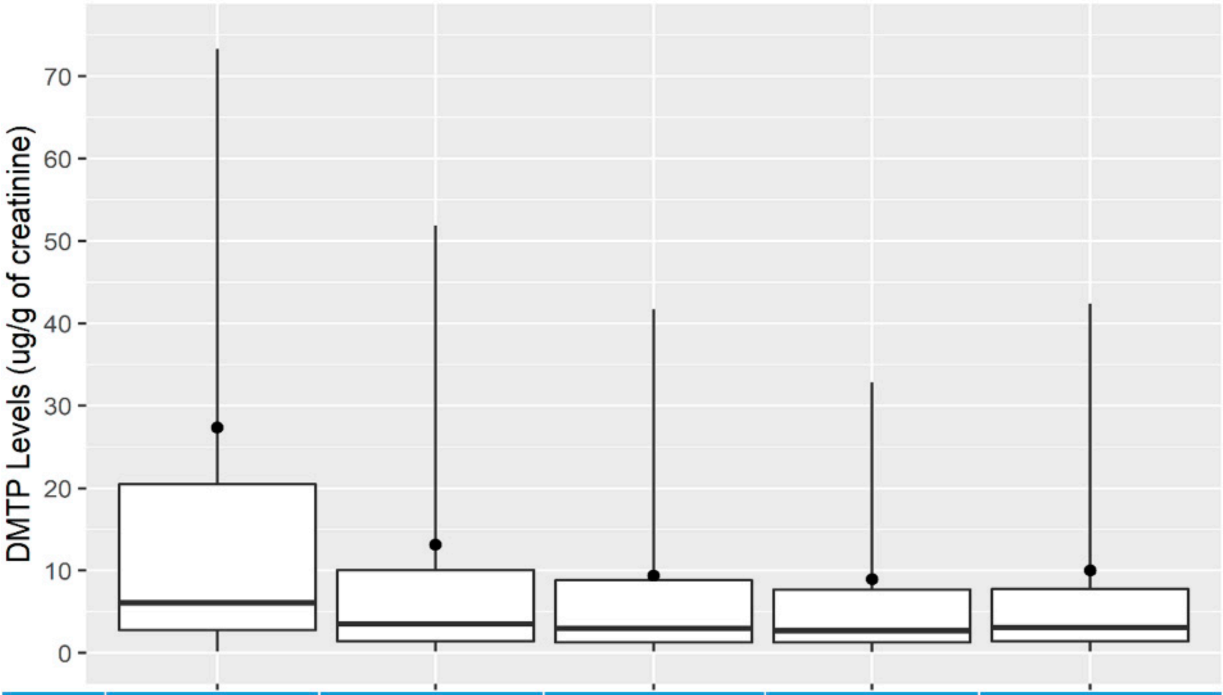

	1999-2000	2001-2002	2003-2004	2005-2006	2007-2008
Percent Above DL	60.17%	47.45%	78.96%	72.23%	74.47%
Maximum	2372.88	443.24	201.02	294.58	260.50
95th Percentile	73.34	51.88	41.71	32.86	42.36
Mean	27.36	13.15	9.40	8.98	10.05
75th Percentile	20.49	10.06	8.83	7.71	7.79
Median	6.07	3.55	3.01	2.73	3.09
25th Percentile	2.75	1.42	1.27	1.28	1.41
Minimum	0.20	0.21	0.17	0.13	0.19

Boxplots represent the minimum, first quartile, median, third quartile, and 95th percentile values. Mean values are represented by the black circle.

Figure S4. Time trends in urinary DETP metabolites in adults from 1999 to 2008.

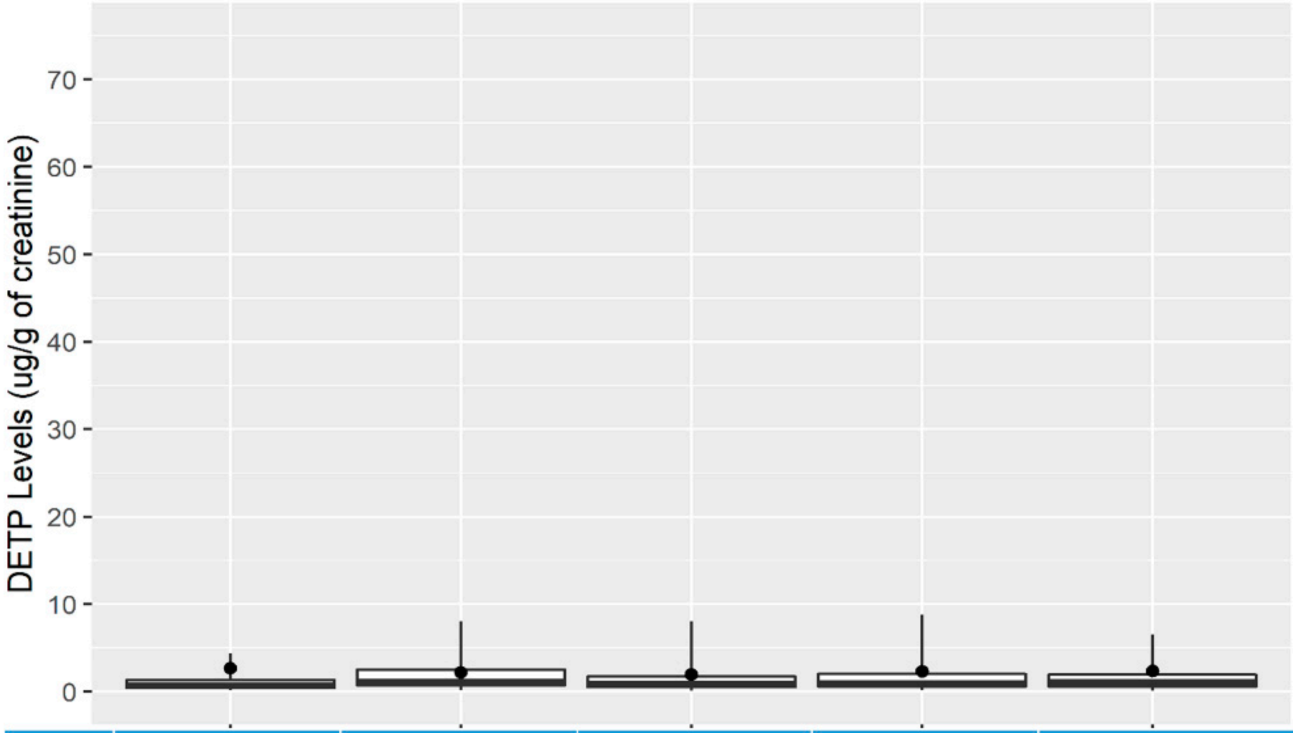

	1999-2000	2001-2002	2003-2004	2005-2006	2007-2008
Percent Above DL	39.94%	53.55%	39.77%	30.44%	41.12%
Maximum	281.82	87.92	33.79	38.65	64.83
95th Percentile	4.35	8.00	8.01	8.81	6.57
Mean	2.68	2.21	1.96	2.33	2.37
75th Percentile	1.36	2.52	1.73	2.02	1.94
Median	0.70	1.19	0.89	0.97	1.10
25th Percentile	0.44	0.72	0.53	0.59	0.61
Minimum	0.16	0.18	0.13	0.17	0.13

Boxplots represent the minimum, first quartile, median, third quartile, and 95th percentile values. Mean values are represented by the black circle.

Figure S5. Time trends in urinary DMDTP metabolites in adults from 1999 to 2008.

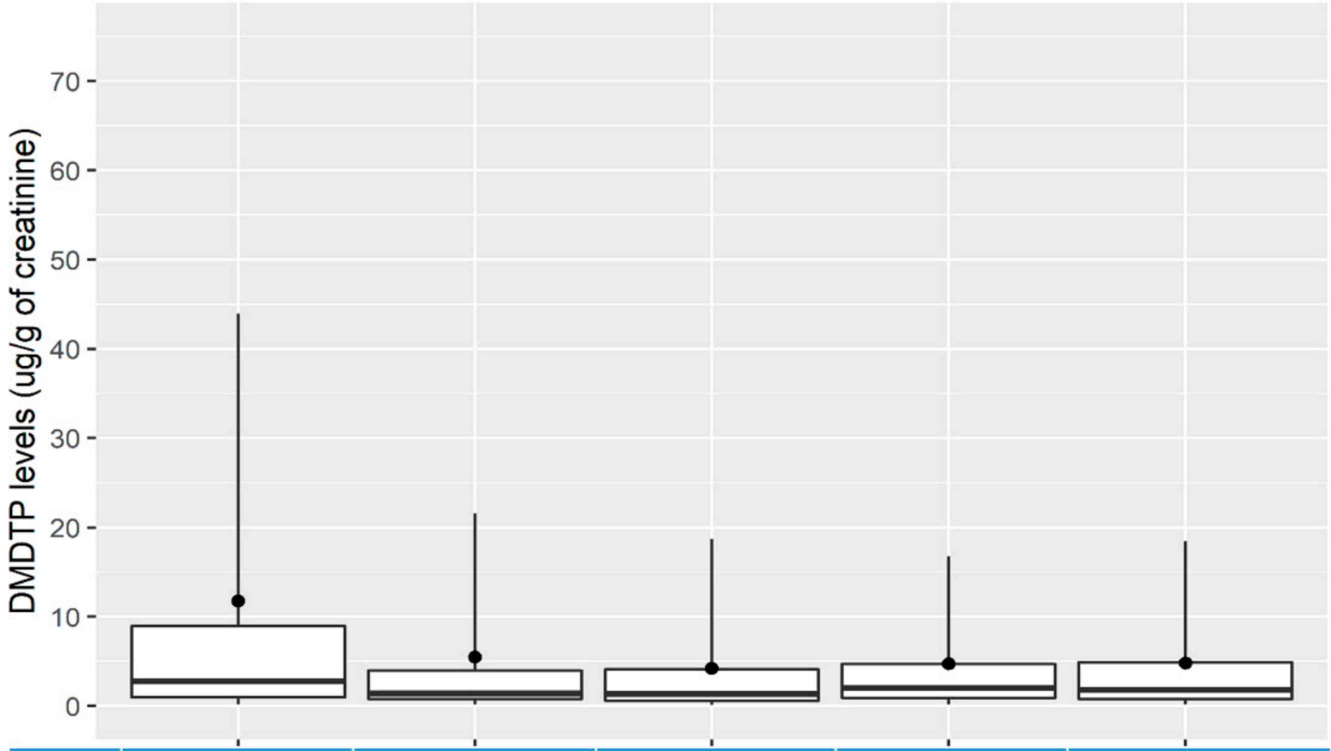

	1999-2000	2001-2002	2003-2004	2005-2006	2007-2008
Percent Above DL	41.91%	28.36%	32.20%	20.64%	22.36%
Maximum	461.54	148.86	76.74	56.19	108.04
95th Percentile	43.96	21.58	18.68	16.75	18.44
Mean	11.77	5.50	4.24	4.73	4.80
75th Percentile	8.98	3.96	4.11	4.69	4.89
Median	2.77	1.44	1.33	2.00	1.80
25th Percentile	0.99	0.79	0.59	0.92	0.79
Minimum	0.15	0.17	0.13	0.16	0.16

Boxplots represent the minimum, first quartile, median, third quartile, and 95th percentile values. Mean values are represented by the black circle.

Figure S6. Time trends in urinary DEDTP metabolites in adults from 1999 to 2008.

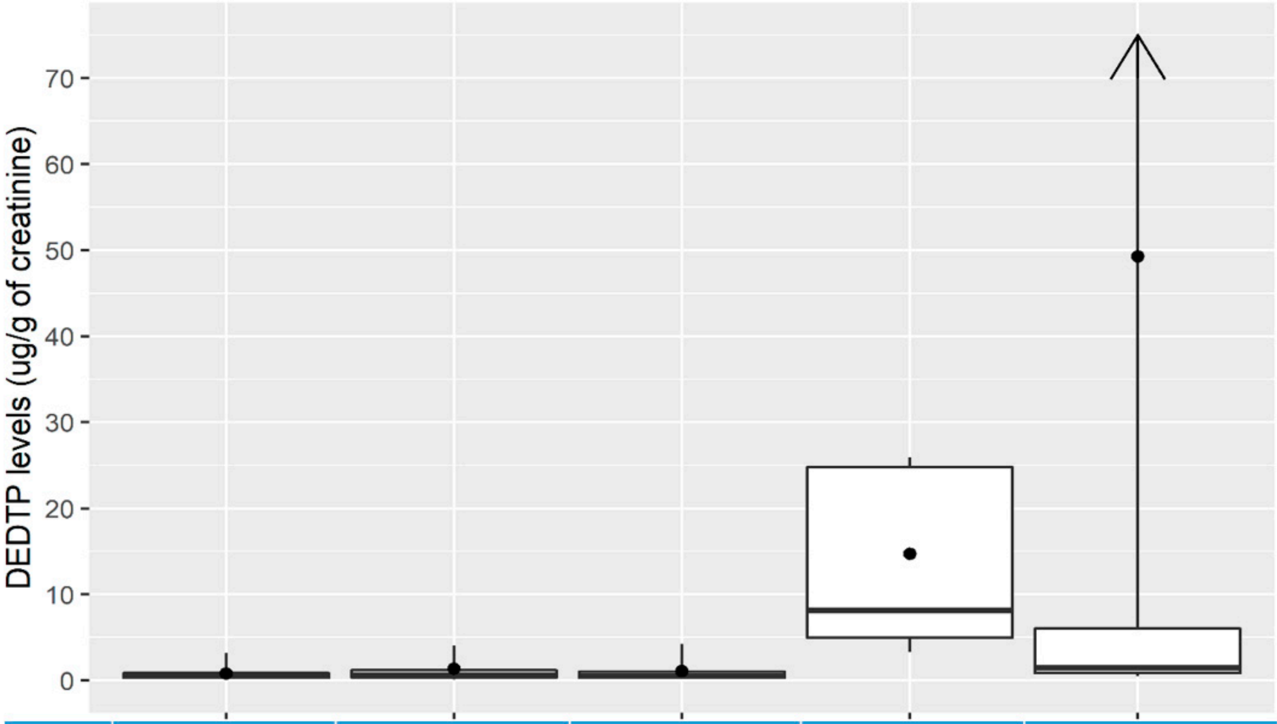

	1999-2000	2001-2002	2003-2004	2005-2006	2007-2008
Percent Above DL	13.17%	16.69%	4.49%	0.37%	0.63%
Maximum	154.55	39.33	10.86	26.21	275.42
95th Percentile	3.17	4.01	4.24	25.93	74.90
Mean	0.83	1.38	1.11	14.75	49.28
75th Percentile	0.87	1.17	1.03	24.80	6.05
Median	0.53	0.64	0.53	8.13	1.48
25th Percentile	0.30	0.35	0.31	4.96	0.86
Minimum	0.09	0.12	0.20	3.28	0.50

Boxplots represent the minimum, first quartile, median, third quartile, and 95th percentile values. Mean values are represented by the black circle.
